# Rating of camera navigation skills in colorectal surgery

**DOI:** 10.1007/s00384-020-03543-9

**Published:** 2020-03-28

**Authors:** F. Huettl, H. Lang, M. Paschold, F. Watzka, N. Wachter, B. Hensel, W. Kneist, Tobias Huber

**Affiliations:** 1 Department of General, Visceral and Transplant Surgery, University Medical Center, Johannes Gutenberg-University Mainz, Langenbeckstraße 1, 55131 Mainz, Germany; 2 Department of General and Visceral Surgery, St. Georg Hospital, Eisenach, Germany

**Keywords:** Camera navigation, Laparoscopic assistance, Global assessment, Surgical education

## Abstract

**Purpose:**

In advanced minimally invasive surgery the laparoscopic camera navigation (LCN) quality can influence the flow of the operation. This study aimed to investigate the applicability of a scoring system for LCN (SALAS score) in colorectal surgery and whether an adequate scoring can be achieved using a specified sequence of the operation.

**Methods:**

The score was assessed by four blinded raters using synchronized video and voice recordings of 20 randomly selected laparoscopic colorectal surgeries (group A: assessment of the entire operation; group B: assessment of the 2nd and 3rd quartile). Experience in LCN was defined as at least 100 assistances in complex laparoscopic procedures.

**Results:**

The surgical teams consisted of three residents, three fellows, and two attendings forming 15 different teams. The ratio between experienced and inexperienced camera assistants was balanced (*n* = 11 vs. *n* = 9). Regarding the total SALAS score, the four raters discriminated between experienced and inexperienced camera assistants, regardless of their group assignment (group A, *p* < 0.05; group B, *p* < 0.05). The score’s interrater variability and reliability were proven with an intraclass correlation coefficient of 0.88. No statistically relevant correlation was achieved between operation time and SALAS score.

**Conclusion:**

This study presents the first intraoperative, objective, and structured assessment of LCN in colorectal surgery. We could demonstrate that the SALAS score is a reliable tool for the assessment of LCN even when only the middle part (50%) of the procedure is analyzed. Construct validity was proven by discriminating between experienced and inexperienced camera assistants.

## Introduction

During laparoscopic surgery, camera navigation is often performed by less experienced members of the surgical team. The limitations of minimally invasive surgery for depth perception, reduction of tactile feedback, and the fulcrum effect (the optical inversion of the movements on the screen) do not apply to the main surgeon only but also to the (camera) assistant [1, 2]. Therefore, handling the laparoscope is a complex psychomotor task that requires an appropriate visual-spatial ability, hand-eye coordination, and anticipatory knowledge of the surgical procedure [1, 3, 4]. Poor camera navigation (i.e., errors in horizon, instrument collisions, smudges, and failure to achieve a stable view) can compromise the flow of the operation resulting in increased frustration of operating surgeons and prolongation of operating time and therefore may compromise patient safety [5, 6].

The importance of high-quality camera assistance has come more into focus over the last years. Several studies were conducted to assess and improve laparoscopic camera navigation, though all these studies were conducted with virtual reality simulators or box trainers [4, 6–9]. With the structured assessment of laparoscopic camera navigation skills (SALAS) score, we developed the first objective intraoperative assessment tool for laparoscopic camera assistance [10]. Although the score was developed using a variety of laparoscopic procedures, construct validity has so far been proven for laparoscopic cholecystectomy, a basic laparoscopic operation. Laparoscopic large bowel procedures qualify as advanced laparoscopic operations according to the Society of American Gastrointestinal and Endoscopic Surgeons (SAGES), suggesting advanced camera navigation as well [11]. The aim of this study was to prove the applicability of the SALAS score to advanced colorectal surgery and to investigate whether adequate scoring can also be achieved using a specified sequence of the operation.

## Materials and methods

### Study design

Twenty randomly selected laparoscopic colorectal resections involving at least a partial mobilization of the left colon (left-sided colonic, sigmoid, or rectum resection) were included in the study.

Video and audio recordings of the surgical team and the endoscope were anonymized for later analysis. The video recording excluded the faces of the surgical team to optimize anonymization of the participating surgeons. For better comparability between the different operations, video recording was started after trocar placement and stopped after high tie ligation of the inferior mesenteric artery (IMA) and finishing of the mesocolic excision. The total mesorectal excision in the case of low rectal cancer was excluded for better comparability. The data collection included also type of resection, operation time, and educational level of the camera assistant. Camera experience was defined as at least 100 assistances (camera navigation) in complex laparoscopic procedures.

### Participants

Participants were surgical residents, fellows, and attending physicians of our department. The participants were allowed to stop the data collection during surgery at any point and without cause.

### Equipment

The intraoperative laparoscopic video was recorded using the available laparoscope (Karl Storz SE & Co KG, Tuttlingen, Germany). For intraoperative video and voice recording of the surgical team, a separate laptop computer (Dell, Round Rock, Texas, USA) connected to a webcam (Logitech, Apples, Switzerland) was used.

### Camera navigation assessment (SALAS score)

For the SALAS score (structured assessment of laparoscopic assistant skills), the number of errors regarding the centering of the operational field (centering), the maintenance of correct horizon angle (horizon), and instrument visualization (target out of view) as well as the number of verbal commands (verbal) and manual corrections (manual) made by the surgeon were analyzed as previously described [10]. The assessment of the score was performed by four trained members of our surgical department who were blinded regarding the identity of the operation team members as well as the date of operation. For the score calculation, the error counts were set in relation to the operation time, resulting in a total SALAS score from 5 (minimum) to 25 (maximum). The raters were divided into two groups, assessing the entire video (group A) or assessing the 2nd and 3rd quartile of the video (group B).

### Statistical analysis

All statistical analyses were conducted using IBM SPSS Statistics 23 (IBM, Armonk, NY, USA). The score’s construct validity was verified using the non-parametric Mann-Whitney *U* test. Intraclass correlation coefficients (ICCs) were used to assess the score’s interrater variability and reliability. Spearman’s rank correlation coefficient was used for correlation analysis. Statistical significance was set at *p* < 0.05 for all comparisons.

### Ethics

The study was approved by the Ethics Committee of the Medical Association of Rhineland-Palatinate, Germany. All participants gave informed consent.

## Results

### Demographics

The participants consisted of three surgical residents, three fellows, and two attending physicians forming 15 different surgical teams in the 20 selected operations. The ratio of operations with experienced and inexperienced camera assistants was balanced (*n* = 11 vs. *n* = 9).

The majority of patients were male (*n* = 15). Mean age was 63.5 years (± 14.2). Table 1 gives an overview of the different underlying diseases and performed surgical procedures. Malignancy occurred in 55% of cases (*n* = 11).

**Table 1:** Characteristics of analyzed procedures (*n* = 20)

Characteristics	Number of procedures (%)
Underlying disease	
Sigmoid diverticulitis	9 (45)
Colon cancer	7 (35)
Rectal cancer	4 (20)
Type of resection	
Sigmoid resection	8 (40)
Sigmoid resection (CME)	2 (10)
Left-sided hemicolectomy (CME)	6 (30)
Low anterior rectal resection (TME)	4 (20)
Educational level of the present camera assistant	
Resident	7 (35)
Fellow	8 (40)
Attending	5 (25)

*CME*, complete mesocolic excision; *TME*, total mesorectal excision

### Camera navigation

**Fig. 1:**
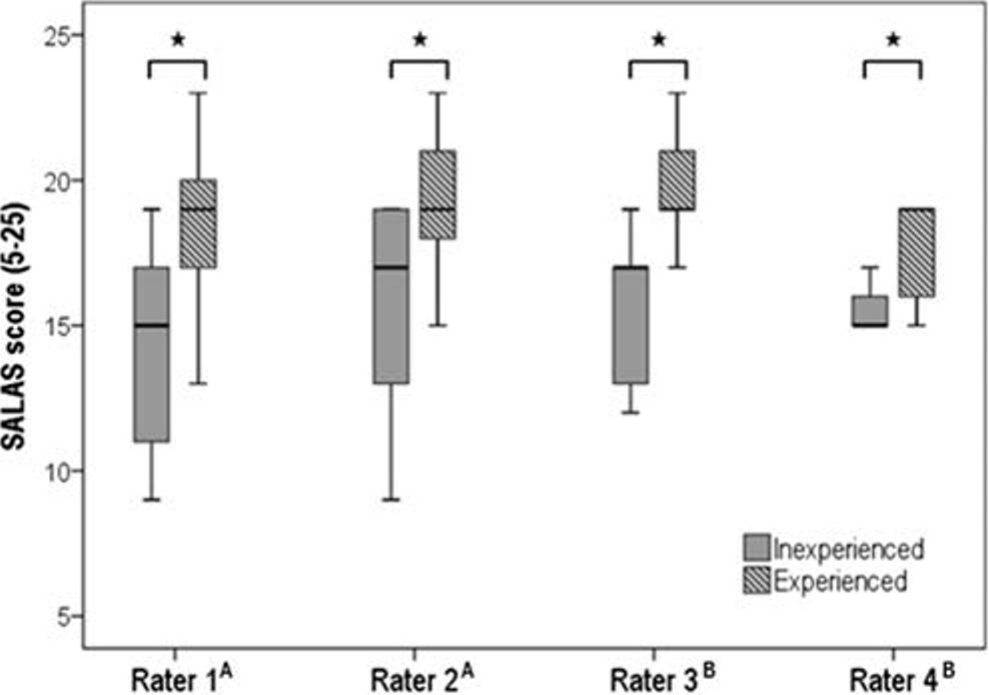
Raters’ assessment of the SALAS score by experienced and inexperienced camera assistants. Significant discrimination (Mann-Whitney *U* test) is marked with a star. Superscript A indicates assessment of the entire video, and superscript B indicates assessment of the 2nd and 3rd quartile of the video

All raters regardless of their group assignment (group A: entire video vs. group B: 2nd and 3rd quartile of video) discriminated significantly between experienced and inexperienced camera assistants with *p*_1_ = 0.007, *p*_2_ = 0.020, *p*_3_ = 0.004, and *p*_4_ = 0.016 (Fig. 1). The score’s interrater variability and reliability were proven with an intraclass correlation coefficient of 0.88 between the four different raters. No statistically relevant correlation was achieved between operation time and SALAS score (*p* > 0.05).

## Discussion

Laparoscopic colorectal surgery requires advanced skills of the whole surgical team. The influence of high-quality camera navigation as an aspect of effective and safe surgery has been shown [5, 12, 13]. The transfer of quality control and quality assessment from simulation-based training into the operation room, however, is not established yet. In addition, it is shown that laparoscopic operative skills are not a precise predictor for the quality of camera navigation; hence, an independent assessment of camera navigation skills is essential for objective quality control [7, 14]. With this study, we present the first intraoperative, structured assessment of camera navigation quality in advanced laparoscopic colorectal surgery.

There has been a growing concern among surgical educators about the surgical competence of residents at the end of their training. Therefore, a competency-based performance assessment for surgical skills has been of increasing interest over the last years [15–17]. For surgical performance, these assessment tools are already part of surgical curricula: OSATS (Objective Structured Assessment of Technical Skills), GOALS (Global Operative Assessment of Laparoscopic Skills), FLS (Fundamentals of Laparoscopic Surgery), but they are still missing for camera navigation. Currently, the SALAS score and the OSA-CNS (Objective Structured Assessment of Camera Navigation Skills) score by Nilsson et al. are the only structured intraoperative assessment tools for camera navigation described in literature. The construct validity of the OSA-CNS was proven on laparoscopic cholecystectomy as a transfer test at the end of a simulation-based training [6]. For the OSA-CNS, the main surgeon, however, assesses the camera assistant’s skills compromising the objectivity of the tool (observer bias) [18]. Moreover, the authors did not report on the score’s intraclass correlation coefficients, debatably compromising the score’s reliability [10]. With the current and previous studies, we could demonstrate high intraclass correlation coefficients (ICC) for the SALAS score in laparoscopic surgery. In addition, construct validity was proven not only for basic but also for advanced laparoscopic procedures. Due to the complexity of precise camera movement and sophisticated use of the camera angle in advanced laparoscopic surgery, the verification of the score’s reliability and construct validity for advanced laparoscopic surgeries is of utter importance. With the results of the current study, the SALAS score proves to be a suitable and to date the only tool for competency-based assessment of intraoperative camera navigation skills in basic and advanced laparoscopic surgery.

A limitation of this assessment tool is the complexity and the effort of scoring due to video assessment. The analysis of the operation time as a surrogate parameter may seem easier at first glance. The comparison of time, however, can only be an adequate surrogate parameter for the assessment of camera navigation quality in standardized simulation scenarios [3, 8, 14, 19]. It is not as qualified for an intraoperative assessment, in which the procedure time is also influenced by the main surgeon’s operative experience and multiple patient-specific factors [20, 21]. Brackmann et al. [5] state that successful camera navigation relies more on accuracy and precision than on time. In accordance with these studies, there was no statistically relevant correlation between operation time and assessed SALAS score in the current study. The comparison of operating time as a parameter for quality control in laparoscopic camera navigation appears to be not appropriate due to the variety of confounders in non-simulation operation scenarios.

As an additional aspect, the current study considered the increasing economic pressure on efficient time usage. Video-based and therefore objective assessment of surgeries especially advanced procedures can be time-consuming as previously described [5]. We demonstrated significant discrimination between experienced and inexperienced camera assistants using the SALAS score even if just the second and third quartile of the procedure is assessed, resulting in a time saving of 50% (mean time difference of 42 min ± 19 min). This new aspect may increase the usability of the SALAS score as an assessment tool.

In conclusion, this study presents the first intraoperative and objective assessment of camera navigation quality in advanced colorectal surgery. The applied SALAS score proves effective in discriminating between experienced and inexperienced camera assistants. Therefore, the SALAS score is suitable and to date the only tool for competency-based assessment of intraoperative camera navigation skills in basic and advanced laparoscopic surgery.
